# Advantages and Limitations of Different p62-Based Assays for Estimating Autophagic Activity in Drosophila

**DOI:** 10.1371/journal.pone.0044214

**Published:** 2012-08-31

**Authors:** Karolina Pircs, Peter Nagy, Agnes Varga, Zsolt Venkei, Balazs Erdi, Krisztina Hegedus, Gabor Juhasz

**Affiliations:** Department of Anatomy, Cell and Developmental Biology, Eotvos Lorand University, Budapest, Hungary; Brigham and Women’s Hospital, Harvard Medical School, United States of America

## Abstract

Levels of the selective autophagy substrate p62 have been established in recent years as a specific readout for basal autophagic activity. Here we compared different experimental approaches for using this assay in Drosophila larvae. Similar to the more commonly used western blots, quantifying p62 dots in immunostained fat body cells of L3 stage larvae detected a strong accumulation of endogenous p62 aggregates in null mutants for Atg genes and S6K. Importantly, genes whose mutation or silencing results in early stage lethality can only be analyzed by microscopy using clonal analysis. The loss of numerous general housekeeping genes show a phenotype in large-scale screens including autophagy, and the p62 assay was potentially suitable for distinguishing bona fide autophagy regulators from silencing of a DNA polymerase subunit or a ribosomal gene that likely has a non-specific effect on autophagy. p62 accumulation upon RNAi silencing of known autophagy regulators was dependent on the duration of the knockdown effect, unlike in the case of starvation-induced autophagy. The endogenous p62 assay was more sensitive than a constitutively overexpressed p62-GFP reporter, which showed self-aggregation and large-scale accumulation even in control cells. We recommend western blots for following the conversion of overexpressed p62-GFP reporters to estimate autophagic activity if sample collection from mutant larvae or adults is possible. In addition, we also showed that overexpressed p62 or Atg8 reporters can strongly influence the phenotypes of each other, potentially giving rise to false or contradicting results. Overexpressed p62 aggregates also incorporated Atg8 reporter molecules that might lead to a wrong conclusion of strongly enhanced autophagy, whereas expression of an Atg8 reporter transgene rescued the inhibitory effect of a dominant-negative Atg4 mutant on basal and starvation-induced autophagy.

## Introduction

Human p62/SQSTM1 (sequestosome-1) is a multidomain scaffold protein involved in various signaling pathways regulating a number of processes including apoptosis, stress responses, and cell growth. [1] Its single Drosophila homolog is also known as Ref(2)P (refractory to sigma P), based on its implicated roles in sigma rhabdovirus multiplication. [2] For simplicity, hereafter we refer to the Drosophila gene as p62. Its encoded protein product shows a similar domain structure to human p62, both containing an N-terminal PB1 domain required for self-oligomerization and binding to other PB1-domain proteins, a ZZ-type zinc finger domain, an LIR (LC3-interacting region) required for its interaction with Atg8/LC3 family members, and a C-terminal ubiquitin-binding UBA (ubiquitin-associated) domain. [3] The Atg8/LC3 interaction enables selective degradation of p62 by autophagy, and by acting as a specific adaptor protein it also ensures the targeting of ubiquitinated proteins for lysosomal degradation. Numerous human degenerative disorders are accompanied by the formation of cytoplasmic aggregates containing p62 and ubiquitinated proteins. [4] These abnormal aggregates also form in response to impaired autophagy, so p62 levels are thought to inversely correlate with dysregulation of basal autophagy. [5], [6].

Different p62-based assays have been implemented in recent years in various experimental systems, including immunostaining of cultured cells or tissue samples, western blots, and GFP-tagged reporters. [7] Drosophila melanogaster is a key in vivo model organism, traditionally used as a tool for the discovery of genes and genetic interactions. Previous reports have already shown the progressive formation of p62/ubiquitin aggregates in adult brains, with greatly enhanced rates upon genetic inhibition of autophagy and in fly models of neurodegenerative disorders. [3], [4], [8], [9] Here we set out to test different p62-based assays in the fat body or whole larvae in a quantitative manner, comparing the results and relative efficiency of the various experimental approaches. Our work has important implications for other model systems as well: we show that I. statistical analysis of p62-positive aggregates in immunostained cells and tissues is similarly effective as western blots for the estimation of basal autophagy levels, II. immunostaining of mosaic fat bodies allows for testing the specific role of genes with lethal phenotypes in basal autophagy, III. endogenous p62 provides a much more sensitive measure of autophagy levels than a constitutively overexpressed GFP-tagged reporter in microscopy, IV. the duration of efficient RNAi knockdown is very important for the p62 assay unlike in the case of induced autophagy, V. p62 and Atg8 reporters may strongly interact with each other, and hence „autophagy phenotypes”.

## Results

**Figure 1:**
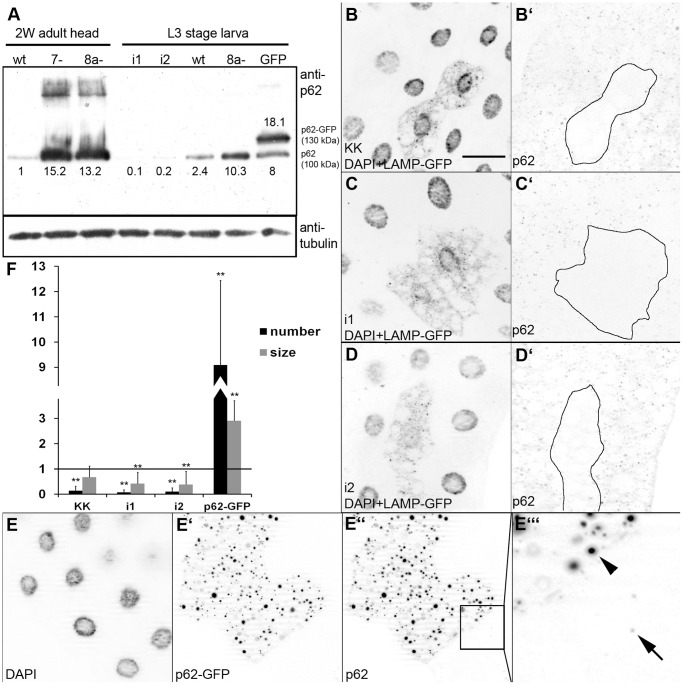
p62 levels in Atg mutants, p62 RNAi and p62 overexpression cells. A. Western blot analysis shows p62 accumulation in Atg8a and Atg7 mutant heads and in Atg8a mutant larvae. RNAi knockdowns of p62 (i1, i2) greatly decrease endogenous protein levels, while overexpression of p62-GFP increases endogenous p62 levels in addition to the appearance of the 130 kDa extra band corresponding to the tagged protein. Numbers refer to p62 protein level relative to tubulin loading control for each sample. B-D. RNAi knockdown of p62 in fat body cell clones (marked by expression of Lamp-GFP) strongly decreases p62 puncta formation. E. Expression of p62-GFP in cell clones increases aggregate formation. Arrowhead in E’ indicates a large aggregate in a p62-GFP expressing cell, arrow marks an endogenous p62 dot in a control cell. F. Statistical evaluation of the number and size of p62 dots for samples in panels B–E. ** indicates a very significant difference (p<0.01), based on two-tailed two-sample unequal Student’s t tests. Scalebar in panel B equals 30 µm for panels B–E. *Genotypes are: (A) lane 1: w [1118], lane 2: Atg7[d77]/Atg7[d14], lane 3: Atg8a[d4], lane 4: UbiGal4/+; p62[HMS00551]/+, lane 5: UbiGal4/+; p62[HMS00938]/+, lane 6: w [1118], lane 7: Atg8a[d4], lane 8: cgGal4/UAS-p62-GFP; (B) hs-Flp; UAS-LampGFP/p62[KK108193]; Act>CD2>Gal4, UAS-Dcr2/+; (C) hs-Flp; UAS-LampGFP/+; Act>CD2>Gal4, UAS-Dcr2/p62[HMS00551]; (D) hs-Flp; UAS-LampGFP/+; Act>CD2>Gal4, UAS-Dcr2/p62[HMS00938]; (E) hsFlp; UAS-p62-GFP/+; Act>CD2>Gal4,UAS-Dcr2/+.*

Given the importance of the p62 assay in characterizing autophagy phenotypes of selected mutants and RNAi treatments, we have generated polyclonal antibodies against endogenous Drosophila p62. The antibody recognized a single prominent band of about 100 kDa on western blots prepared form L3 stage larvae or adult heads (**Figure 1a**). The intensity of this band was reduced more than 10-fold relative to tubulin levels upon silencing of p62 in both cases of ubiquitously expressed different RNAi transgenes in L3 larvae. Null mutation of Atg8a resulted in a substantial increase of p62 levels in larvae and in adult heads, respectively, similar to Atg7 mutant heads. Expression of p62-GFP in the larval fat body gave rise to a slower mobility band of about 130 kDa, corresponding to the tagged version of this protein (**Figure 1a**). Interestingly, the presence of p62-GFP also increased the levels of endogenous p62 3.3-fold on an organismal level (probably much higher in case of the fat body, the tissue in which the tagged protein was expressed). This finding suggests that overexpression of a p62 reporter strongly drives aggregate formation, and that endogenous p62 may also be captured and stabilized in these structures.

Next we analyzed the distribution of p62 in larval fat body cells by immunostaining whole-mount tissues. We found that endogenous p62 formed numerous small cytoplasmic dots in these cells, and RNAi silencing of p62 using three different transgenes all strongly and significantly reduced the number of these dots (**Figure 1b–d**, see **Figure 1f** for statistics). As expected, expression of p62-GFP in fat body cell clones strongly promoted aggregate formation, resulting in a 9-fold increase in the number and 2.8-fold increase in the size of p62-positive structures, respectively (**Figure 1e, f**).

**Figure 2:**
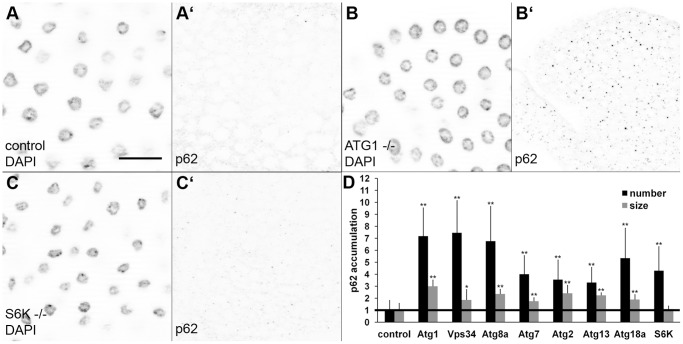
Endogenous p62 accumulates in Atg and S6K mutants. A. p62 immunostaining detects small dots scattered throughout the cytoplasm in fat body cells of wild-type larvae. B. p62 aggregates appear bigger and more numerous in Atg1 null mutants, while loss of S6K (C) only increases the number, but not the size of p62 aggregates. D. Statistical evaluation of p62 puncta in fat bodies of various Atg mutants and in S6K null animals. * indicates a significant difference (p<0.05), ** indicates a very significant difference (p<0.01), based on two-tailed two-sample unequal Student’s t tests. Scalebar in panel A equals 30 µm for panels A–C. *Genotypes are: (A) w [1118]; (B) Atg1 [25]; (C) S6K[l–1].*

Since defective basal autophagy is known to enhance p62 aggregation, we analyzed previously described null mutants for selected genes involved in autophagy. Both the number and size of p62-positive dots were significantly increased in fat bodies dissected from L3 stage larvae mutant for the following core autophagy genes: Atg1 and Atg13 (encoding members of the Atg1 kinase complex), Vps34 (encoding the phosphatidyl-inositol 3-kinase), Atg2 and Atg18a (encoding binding partners of the transmembrane protein Atg9), Atg7 and Atg8a (encoding the E1-like enzyme and one of the ubiquitin-like proteins in the autophagy-specific protein conjugation systems) (**Figure 2a, b, d**, see also Figure S1 for additional images). S6 kinase is a physiologic substrate of TOR kinase (Target Of Rapamycin), a central regulator of autophagy and cell growth. Activation of TOR suppresses autophagy and results in phosphorylation of S6K. Based on these correlations, S6K was long considered as an inhibitor of autophagy. Therefore, the finding that Drosophila S6K was actually required for starvation-induced autophagy was surprising. [10], [11] In line with those observations, we have also found that loss of S6K significantly increased the number (but in this case not the size) of p62 aggregates in larval fat body cells, consistent with its suggested positive role in autophagy (**Figure 2c**, compare to **Figure 2a**; see also **Figure 2d** for quantification).

**Figure 3:**
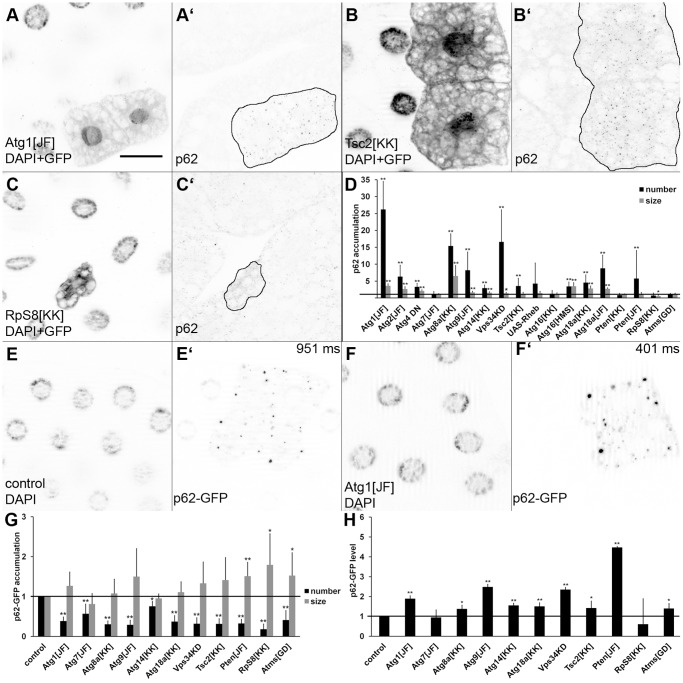
Loss of known autophagy regulators enhances p62 puncta formation cell-autonomously. Knockdown of Atg1 (A) or Tsc2 (B) increases p62 aggregate formation, while silencing of RpS8 results in a slight reduction of p62 dot number (C). Panel D shows statistical evaluation of the effect of RNAi and overexpression lines on p62 accumulation. Overexpressed p62-GFP forms multiple aggregates in control and Atg1 RNAi cells (panels E and F, respectively). Exposure times are indicated in the top right corner for panels E and F. Statistical evaluation of p62-GFP aggregate size and number in various RNAi and overexpression lines reveals changes that are difficult to interpret (G), but p62-GFP levels inferred from exposure times during image acquisition are qualitatively similar to data obtained with anti-p62 immunostaining (compare H to D). * indicates a significant difference (p<0.05), ** indicates a very significant difference (p<0.01), based on two-tailed two-sample unequal Student’s t tests in panels D, G, H. Scalebar in panel A equals 30 µm for panels A–C and E–F. *Genotypes are: (A) hsFlp; UAS-Dcr2/+; Act>CD2>Gal4, UAS-GFPnls/Atg1[JF02273]; (B) hsFlp; UAS-Dcr2/TSC2[KK103417]; Act>CD2>Gal4, UAS-GFPnls/+; (C) hsFlp; UAS-Dcr2/RpS8[KK106835]; Act>CD2>Gal4, UAS-GFPnls/+; (E) hsFlp; UAS-p62-GFP/+; Act>CD2>Gal4, UAS-Dcr2/+ (F) hsFlp; UAS-p62-GFP/+; Act>CD2>Gal4, UAS-Dcr2/Atg1[JF02273].*

We then evaluated how inactivation of known regulators of autophagy in somatic clones of mosaic animals affected p62 levels. RNAi silencing of Atg1 or Atg14 (the autophagy-specific subunit of the Vps34 complex) enhanced p62 aggregation, similar to overexpression of dominant-negative Vps34 (**Figure 3a, d**; see also Figure S2). Silencing of Atg2, Atg9 and Atg8a also promoted p62 dot formation. Enhancing TOR signaling by overexpression of its activator Rheb or knockdown of the Rheb inhibitor Tsc2 also increased p62 dot number, although in case of Rheb the phenotypes were so variable that the change was not statistically significant (**Figure 3b, d**; see also Figure S2). Silencing of Atg7 did not lead to increased p62 dots, consistent with the observation that this line had a weaker effect on starvation-induced autophagy relative to the other lines we used, potentially caused by either less efficient knockdown or by the kinetics of the Atg7 E1-like enzyme reaction, that is, lowered levels of Atg7 due to RNAi being potentially sufficient to support basal autophagy (data not shown).

Numerous genes are required for fundamental cellular processes such as transcription or translation. It is not so surprising that these genes are often identified as putative hits in various genome-wide RNAi screens, although their effect is likely indirect in most cases. While knockdown of many of these genes reduces cell size to an extent that evaluation of autophagy phenotypes becomes practically impossible, silencing of the genes coding for the RNA polymerase II. complex member Atms or the ribosomal subunit RpS8 strongly interfered with starvation-induced mCherry-Atg8 and Lysotracker puncta formation (Figure S3). On the other hand, these knockdowns did not enhance p62 aggregation as expected from a condition that indirectly interferes with autophagy induction through perturbing a fundamental cellular process and/or the expression of a transgenic marker (**Figure 3c, d**; see also Figure S2), suggesting that the p62 assay is potentially suitable for distinguishing specific regulators of autophagy (“hits”) from indirect effects (“noise”).

A GFP-tagged p62 reporter was previously used successfully in Drosophila to detect basal autophagy defects in Atg13 mutant cells. [12] We decided to investigate how phenotypes obtained by p62-GFP compare with those based on the endogenous protein. Expression of p62-GFP together with RNAi against Atg1, Atg7, Atg8a, Atg9, Atg14, Atg18a, Tsc2, Pten or with dominant-negative Vps34 were all found to decrease the number of GFP-positive aggregates (**Figure 3e–g**; see also Figure S4). In case of Pten and Tsc2 the size of GFP-positive aggregates increased significantly, while it did not change in most cases. Importantly, the number of these aggregates also decreased while their size increased in case of both RpS8 and Atms. As no clear conclusions could be drawn based on these results, we also looked at exposure times used during image acquisition. With this approach we could detect significantly increased GFP signal for Atg1, Atg8a, Atg9, Atg14, Atg18a, Pten RNAi and Vps34KD expressing cells, but not in the case of Atg7, Tsc2, RpS8 and Atms RNAi cells (**Figure 3e–h**; see also Figure S4). These results well agreed with the data obtained with the endogenous p62 antibody differing only in the case of Tsc2, although there was a several-fold difference for each positively scoring line in favor of the endogenous p62 assay.

**Figure 4:**
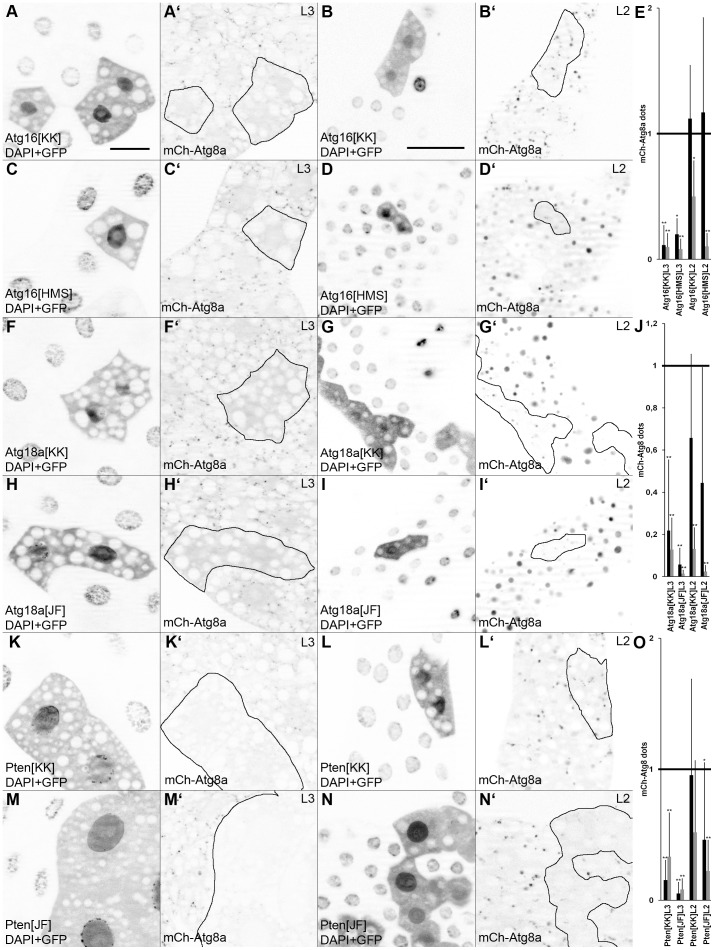
The effect of Atg16, Atg18a and Pten RNAi lines on starvation-induced autophagy in L3 and L2 larval stages. Both RNAi lines for Atg16 show a similar block of mCherry-Atg8a puncta formation in L3 (compare A to C), while the size of these dots is reduced more efficiently by Atg16^HMS^ in L2 (compare B to D; see also panel E for statistics). Both RNAi lines for Atg18a strongly inhibit mCherry-Atg8a dot formation in L3 (compare F to H), while Atg18a^JF^ shows a more complete block in L2, reducing both the size and number of puncta (compare G to I; see also panel J for statistics). Both RNAi lines for Pten strongly inhibit mCherry-Atg8a dot formation in L3 (compare K to M), while Pten^JF^ shows a more complete block in L2, again reducing both the size and number of puncta (compare L to N; see also panel O for statistics). * indicates a significant difference (p<0.05), ** indicates a very significant difference (p<0.01), based on two-tailed two-sample unequal Student’st tests in panels E, J, O. Scalebar in panel A equals 30 µm for panels A, C, F, H, K, M, and scalebar in panel B equals 30 µm for panels B, D, G, I, L, N. *Genotypes are: (A, B) hsFlp; UAS-Dcr2/Atg16[KK105993]; Act>CD2>Gal4, UAS-GFPnls, r4-mCherry-Atg8a/+; (C, D) hsFlp; UAS-Dcr2/+; Act>CD2>Gal4, UAS-GFPnls, r4-mCherry-Atg8a/Atg16[HMS01347]; (F, G) hsFlp; UAS-Dcr2/Atg18a[KK105366]; Act>CD2>Gal4, UAS-GFPnls, r4-mCherry-Atg8a/+; (H, I) hsFlp; UAS-Dcr2/+; Act>CD2>Gal4, UAS-GFPnls, r4-mCherry-Atg8a/Atg18a[JF02898]; (K, L) hsFlp; UAS-Dcr2/Pten[KK101475]; Act>CD2>Gal4, UAS-GFPnls, r4-mCherry-Atg8a/+; (M, N) hsFlp; UAS-Dcr2/+; Act>CD2>Gal4, UAS-GFPnls, r4-mCherry-Atg8a/Pten[JF01987].*

While testing a large number of RNAi lines in our genome-wide RNAi screen (to be described elsewhere), one striking observation was that the p62 assay occasionally showed large differences in case of two different RNAi lines targeting the same gene. For example, two different transgenic RNAi lines for Atg18a had quantitatively different effects on p62 aggregation, potentially reflecting differences in knockdown efficiencies. Interestingly, one of two different RNAi lines against Atg16 (encoding a protein required for Atg8a lipidation) failed to enhance p62 aggregation, similar to the case of PTEN, inactivation of which strongly enhances insulin signaling to promote cell growth and suppress autophagy (**Figure 3d**; see also Figure S2). As both RNAi lines for Atg18a, Atg16 and PTEN very effectively blocked starvation-induced autophagy in L3 larvae, we decided to look for potential reasons of these discrepancies. Quantification of mCherry-Atg8a dots in starved fat bodies revealed that Atg18a^JF^ showed better suppression than Atg18a^KK^ in both L3 and L2 stage animals, likely explaining the quantitatively different p62 results (**Figure 4f–j**). In case of PTEN RNAi lines (**Figure 4k–o**), both showed a strong suppression of mCherry-Atg8a dot formation in L3 stage, while PTEN^KK^ did not significantly inhibit starvation-induced autophagy in L2 animals, consistent with its failure to enhance p62 dot formation. Interestingly, no differences could be seen in starved L3 stage animals between the two RNAi lines in case of Atg16. However, Atg16^HMS^ reduced the size of dots 8-fold, while Atg16^KK^ showed only a two-fold reduction in L2 stage (**Figure 4a–e**). Larger mCherry-Atg8a dots are thought to represent mature autolysosomes, and Atg16^HMS^ had a stronger block on formation of these structures in L2 stage larvae. Lysotracker stainings further supported this hypothesis, as only Atg16^HMS^ reduced the size of Lysotracker puncta statistically significantly in L2 stage animals (Figure S5). Altogether, these results suggest that differences seen in starvation-induced autophagy in the L2 stage and the progressive formation of p62 aggregates (analyzed in the L3 stage) may both reflect the knockdown efficiency of the given RNAi line in earlier stages (that is, L2).

**Figure 5:**
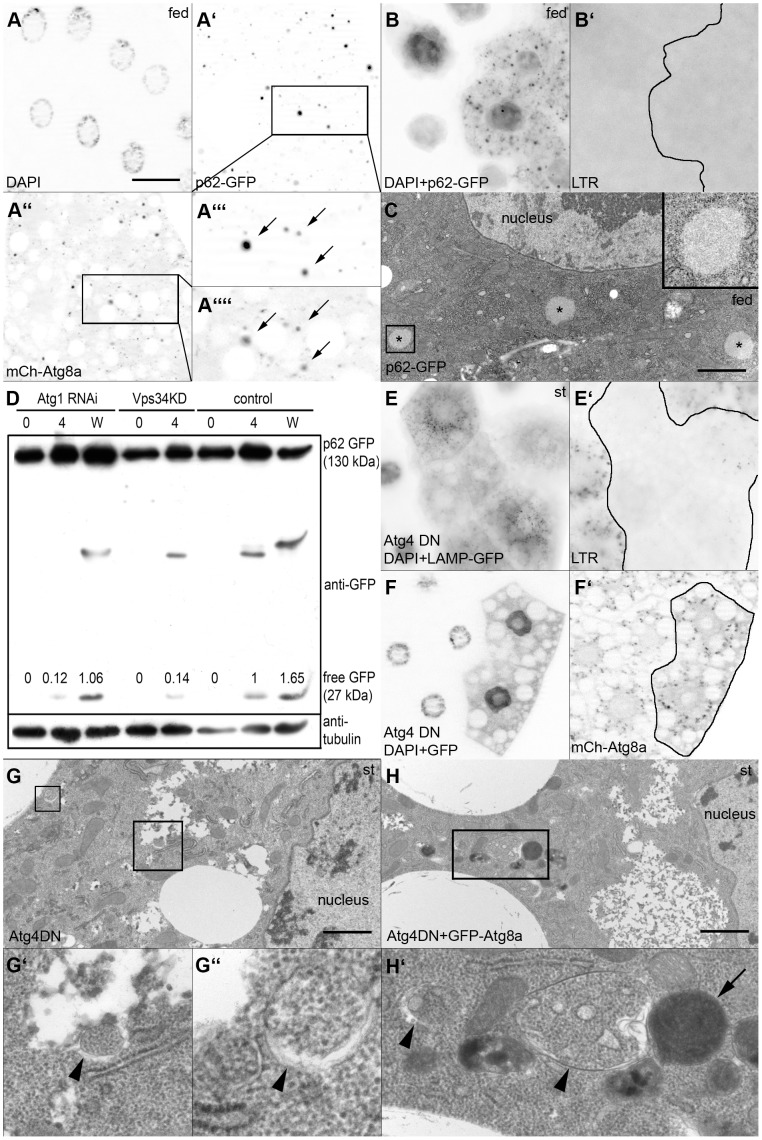
The effect of p62 and Atg8a reporters on autophagy phenotypes. A. Coexpression of p62-GFP and mCherry-Atg8a results in formation of aggregates containing both tagged proteins in fed animals, potentially suggesting enhanced autophagy. B. Expression of p62-GFP fails to induce Lysotracker puncta in fed animals. C. p62-GFP expression leads to the appearance of protein aggregates in ultrastructural images of fat body cells (asterisks), but no autophagic structures are seen in the cytoplasm. Inset shows an enlarged aggregate to illustrate that these inclusion bodies are not membrane-bound. D. Western blot analysis using anti-GFP antibodies of larval extracts expressing mCherry-Atg8a and p62-GFP in the fat body. No generation of free GFP by autolysosomal degradation is seen in well-fed control larvae, it is only induced by a 4-hour starvation or in the wandering stage. Silencing of Atg1 or expression of dominant-negative Vps34 inhibits autophagy-mediated conversion of p62-GFP to free GFP. E, F. Expression of dominant-negative Atg4 blocks starvation-induced Lysotracker puncta formation, but it has no effect on overexpressed mCherry-Atg8a dots. G. Ultrastructural analysis of starved fat bodies reveal small autophagosomes (marked by arrowheads in G’ and G’), but no autolysosomes are seen. Coexpression of GFP-Atg8a with dominant-negative Atg4 rescues this inhibition: numerous autolysosomes (arrow) and autophagosomes (arrowheads) are seen in H, H’. Scalebar in panel A equals 30 µm for panels A, B, E, F and scalebars in panels C, G, H equal 2 µm. *Genotypes are: (A) hsFlp; UAS-p62-GFP/+; Act>CD2>Gal4, UAS-Dcr2/r4-mCherry-Atg8a; (B, C) hsFlp; UAS-p62-GFP/+; Act>CD2>Gal4, UAS-Dcr2/+; (D) lanes 1, 2, 3: cgGal4, UAS-p62-GFP/+; Atg1[JF02273]/+, lanes 4, 5, : cgGal4, UAS-Vps34[KD]/UAS-p62-GFP, lanes 6, 7, 8, : cgGal4, UAS-p62-GFP/+; (E, G) hs-Flp; UAS-Dcr2/+; Act>CD2>Gal4, UAS-GFPnls/UAS-Atg4[C98A]; (F, H) hs-Flp; UAS-Dcr2/+; Act>CD2>Gal4, UAS-GFPnls, r4-mCherry-Atg8a/UAS-Atg4[C98A].*

Finally, we have evaluated the impact of overexpressed p62 and Atg8a reporters (which potentially create a gain of function condition) on autophagy phenotypes, as the possibility of genetic interactions could not be excluded. In fact, when p62-GFP was co-expressed with mCherry-Atg8a, large colocalizing GFP- and mCherry-positive aggregates were formed even in well-fed animals (**Figure 5a**). These aggregates were likely caused by the specific interaction of the two overexpressed proteins and represented an artefact and not bona fide autophagic structures, as Atg8a strongly binds to the LC3-interacting region of p62. In line with that, we did not detect an obvious increase in autophagy in p62-GFP expressing cells by Lysotracker staining and transmission electron microscopy (**Figure 5b, c**). Similarly, we saw no conversion of p62-GFP to free GFP in western blots of larvae coexpressing mCherry-Atg8a and p62-GFP in the fat body, suggesting that mCherry-Atg8a expression does not obviously enhance autophagic degradation of p62-GFP in well-fed L3 stage control animals. As expected, free GFP was readily detected after a 4-hour starvation or in wandering stage animals undergoing developmental autophagy. Expression of Atg1 RNAi or dominant-negative Vps34 strongly decreased starvation-induced conversion of p62-GFP, while Atg1 silencing showed only a weaker effect in wandering animals (**Figure 5d**).

Atg4 encodes a cysteine protease required for the cleavage of the C-terminal amino acids of Atg8a, which is necessary for its subsequent activation and conjugation to the membrane lipid phosphatidyl-ethanolamine. Atg8a needs to be delipidated after successful completion of an autophagosome to allow lysosomal fusion. A mutant form of human Atg4B with a substitution of cysteine 74 to alanine was shown to act in a dominant-negative fashion, inhibiting proper formation and clearence of autophagosomes. [13] We reconstituted this mutant in Drosophila, creating transgenic flies with inducible expression of Atg4a^C98A^. Expression of dominant-negative Atg4a strongly suppressed Lysotracker staining in fat body cells of starved larvae, and also caused accumulation of p62 (**Figure 5e** **,** **Figure 3d**; see also Figure S2). As expression of mCherry-Atg8a in Atg8a null mutants could rescue the p62 accumulation phenotype (Figure S6a, b), we tested whether overexpressed mCherry-Atg8a can also rescue the effect of dominant-negative Atg4. Co-expression of mCherry-Atg8a with dominant-negative Atg4 restored both starvation-induced autophagy in starved animals and the normal turnover of p62 (**Figure 5f**; see also Figure S6c, d). Ultrastructural analysis further confirmed that expression of dominant-negative Atg4 strongly inhibited starvation-induced autophagy: no autolysosomes were seen in cross-sections of fat body cells, while small autophagosome-like structures were occasionally observed (**Figure 5g**). Coexpression of Atg8a with dominant-negative Atg4 restored starvation-induced autophagy, as numerous autolysosomes and autophagosomes were seen in all cells (**Figure 5h**), similar to previous findings in mammalian cells. [13] Similarly, knockdown of Atg4a using the RNAi line Atg4a^KK^ blocked starvation-induced Lysotracker staining and induced p62 accumulation in fat body cells but failed to inhibit mCherry-Atg8a dot formation (not shown), further supporting our hypothesis that partial loss of Atg4a function can be rescued by overexpression of Atg8a.

## Discussion

Determining the levels of the specific autophagy substrate p62 is becoming a standard assay to estimate basal autophagy rates. [7] Western blots are the most widely used experimental setup to detect endogenous p62 levels with the possibility to distinguish soluble and aggregated forms using differential detergent extractions, but these experiments are simply not feasible in studies that involve genetic mosaic animals. Here we presented evidence that an immunostaining approach is similarly effective as western blots to detect even subtle changes of p62 aggregation in the larval fat body, and it can actually provide additional information compared with western blots, as the number, size and intracellular distribution of these aggregates are all detected this way. We recommend using a p62-specific antibody rather than a constitutively overexpressed GFP-tagged reporter as the latter led to extensive self-aggregation and also increased the level of the endogenous protein, because of which the size and number of p62 aggregates were no longer indicative of autophagic activity. Still, comparing GFP signal intensities may yield qualitatively similar information to data obtained by looking at endogenous p62 levels in some but not all cases. Endogenous p62 protein levels may also be influenced by changes unrelated to autophagy, which should not be a problem with a tagged reporter expressed from an artificial promoter. In addition to comparing fluorescence intensities of p62-GFP expressing cell clones, a probably more reliable approach is to follow the generation of free GFP produced by autolysosomal degradation in western blots of larvae expressing p62-GFP by a tissue-specific promoter. Alternatively, p62-GFP expression can be induced by a heat shock in mutant animals and the decay of the tagged protein followed in western blots or microscopy to measure tagged p62 half-life in a pulse-chase assay. This a very efficient method to determine flux during basal or starvation-induced autophagy in cultured cells. [7] Regarding endogenous p62 expression, we found that it is transcriptionally upregulated 4.8-fold in larval fat body cells in response to a 4-hour starvation, [14] and a similar starvation-induced upregulation is also obvious on western blots. Interestingly, starvation mainly increases soluble p62 levels and decreases the amount of the aggregated fraction. [4] This observation also suggests that evaluation of endogenous p62 aggregates is a reliable measure of autophagic flux.

In case of autophagy induced by a certain stimulus such as starvation, levels of proteins required for autophagy at the time of the stimulus and during the response are critical determinants of the magnitude of autophagy that follows. In contrast, p62 levels are also strongly influenced by basal autophagic activity, so formation of excess p62 aggregates is a progressive, time-dependent process. Therefore, duration of a certain treatment or effective gene silencing by RNAi has a major impact on this type of assay. In line with that, we have shown here that p62 levels may remain normal despite a strong block of starvation-induced autophagy if the onset of efficient gene knockdown is delayed during larval Drosophila development. Based on these observations, the perdurance of maternally supplied gene products during development may also account for some of the differences seen in p62 levels in different Atg mutants.

Additionally, certain problems may arise when using overexpressed reporter transgenes for autophagy. First, overexpressed Atg8a reporters were found to be captured in overexpressed p62 aggregates, giving a false impression of increased autophagy. Second, overexpression of an Atg8a reporter may genetically rescue the partial loss of a gene as seen in case of Atg4a, again complicating the interpretation of potentially contradicting results. While the examples shown here may be rare, we have seen several instances when inactivation of certain genes led to such a high accumulation of endogenous p62 aggregates that sequestered Atg8a reporter molecules, and overexpressed Atg8a reporters also seemed to genetically rescue the effect of a number of RNAi lines on autophagy.

Finally, although our experiments involved fat body cells of Drosophila larvae, we are convinced that the results and limitations presented here will be applicable to most cells and organisms used in autophagy research.

## Methods

### Fly Husbandry

Drosophila stocks were maintained on a standard cornmeal/sugar/agar medium at 25°C and 50% humidity on a 12 h light/12 h dark cycle. Thestocks used in this study were: *UAS-p62-GFP, Atg13[*Δ*81],* [*12*] *p62[KK108193], Atg8a[KK109654], Atg18a[KK105366], Atg14[KK108559], Atg16[KK105993], TSC2[KK103417], Pten[KK101475], RpS8[KK106835], Atms[GD20876],* [*15*] *Atg7[d77]/Atg7[d14],* [*8*] *Vps34[*Δ*m22], UAS-Vps34[KD],* [*16*] *p62[HMS00551], p62[HMS00938], Atg1[JF02273], Atg18a[JF02898], Atg9[JF02891], Atg7[JF02787], Atg16[HMS01347], Atg2[JF02786], Pten[JF01987],* [*17*] *Atg2[EP3697]/Df(3L)BSC119], Atg18a[KG03090]/Df(3L)Exel6112], S6K[l-1], UAS-Rheb[EP50.084cre(w-)]/TM6B, w [1118],* [*11*] *Atg1 [25].* [*18*]. The *Atg8a[d4]* mutant used in this study was generated by imprecise excision of the P element *KG07569* and harbors a deletion removing the first 25 codons of Atg8a and the first 53 codons of CG1826, a gene with an unknown function (kindly provided by Tom Neufeld). The mutants are homozygous viable and fertile with no gross morphological or developmental abnormalities. *UAS-Atg4[C98A]* transgenics were established as described below.

### Molecular Cloning and Embryo Injections

The full-length coding sequence of Atg4a/CG4428 with a Cysteine 98 to Alanine mutation changing the coding triplet from TGC to GCC was chemically synthesized (Genscript), and cloned into pUAST using EcoRI and XhoI restriction sites. Drosophila embryo transformation was carried out according to standard methods (Bestgene).

### Polyclonal Anti-p62 and Anti-GFP Antibodies and Western Blots

The polyclonal affinity-purified p62 antibody was raised in rabbits using the peptide antigene PRTEDPVTTPRSTQ corresponding to amino acids 297–311 (Genscript). Polyclonal anti-GFP antibodies were raised using standard procedures by immunizing rats with bacterially expressed His-tagged eGFP purified on Ni affinity columns (Sigma). Samples were separated by SDS-PAGE on an 8% acrylamide gel and transferred to Immobilon-P PVDF membrane (Millipore). Membranes were blocked in 3% milk/TBS for 1 h at room temperature and washed three times for 10 min each in TBST (TBS +0.1% Tween-20). Blots were incubated with primary antibodies: rabbit polyclonal anti-p62 [1∶8,000], mouse monoclonal anti-tubulin AA4.3 (DSHB) [1∶200], rat polyclonal anti-GFP [1∶10,000] in 1.5% milk/TBST for 1 h at room temperature, followed by three 10-min washes in TBST. Blots were incubated in AP-conjugated goat anti-rat (Sigma), anti-rabbit or anti-mouse secondary antibody (Millipore) diluted 1∶10,000 in 1.5% milk/TBST for 1 h at room temperature. Blots were washed for 3×10 min in TBST and then incubated with Immobilon Western Chemiluminescent AP Substrate (Millipore), followed by exposure to Super RX film (Fuji).

### Histology and Imaging

Clonal analysis using the spontaneously activated Gal4/UAS system in the larval fat body was carried out as described previously. [11], [12], [16], [19] Bisected third instar larvae were inverted and fixed with 3.7% paraformaldehyde in PBS overnight at 4°C. Next, samples were rinsed twice and washed for 2 hours in PBS, permeabilized for 15 minutes in PBTX-DOC (PBS with 0.1% Triton X-100 and 0.05% sodium deoxycholate) and blocked for 3 h in 3% goat serum in PBTX-DOC. Samples were then incubated overnight at 4°C with primary antibodies rabbit polyclonal anti-p62 [1∶2,000] and mouse monoclonal anti-GFP [1∶1,500] (Invitrogen) in 1% goat serum in PBTX-DOC. After 3×30 minutes washes in PBTX-DOC, samples were incubated with secondary antibodies goat anti-mouse Alexa 488 and goat anti-rabbit Alexa 568 [1∶1,500] (Invitrogen) in 1% goat serum in PBTX-DOC for 4 hours at room temperature. Finally, after 3×15 minutes washes in PBTX-DOC and 1×15 minutes in PBS, fat bodies were dissected and mounted in 50% glycerol/PBS with 0.2 µM DAPI. For p62 staining of mCherry-Atg8a expressing cells Alexa 647-conjugated goat anti-rabbit antibody was used to avoid detection of signal from mCherry. Lysotracker stainings have been carried out as described previously. Images were captured on a Zeiss Axioimager M2 microscope equipped with an Apotome2 grid confocal unit, a Plan-NeoFluar 40×0.75 NA objective, Axiocam Mrm camera, and Axiovision software using a MinMax setting for automatically adjusting image levels. Lysotracker stainings were photographed in widefield mode, and single optical sections are shown for colocalisations and mCherry-Atg8a assays. For p62 stainings, 3 subsequent optical sections taken at 0.55 µm intervals were projected into a single plane using Maximum Intensity Projection.

### Statistical Analysis

For western blots, the image of the scanned film was inverted in Adobe Photoshop, saved and loaded in ImageJ. The strongest band was selected first, and individual bands were measured (Analyze, Set measurements, Integrated density, Measure) using a selection area with the same width and height as for the strongest band. Mean p62 values were normalized to tubulin mean scores first, and then expression levels were calculated relative to the control sample (wt head, lane 1). For immunostainings, images were loaded in ImageJ, and the image type was changed from RGB Color to RGB Stack. In the case of mutants, a 300×300 dpi area was chosen randomly from the red channel, and the threshold was adjusted to select dots. Each selected area was analyzed (Analyze Particles, Show, Masks) and the count and average size (in pixel^2^) were noted from the summary of masked images. At least 10 images were evaluated from 4–6 animals per genotype. The number/size data were summarized in Excel, and normalized to control (average dot number and size in control animals was set to 1). For clonal analysis, image type was again changed from RGB Color to RGB Stack. Then the threshold was adjusted for the adequte channel, and clone cells and neighbouring control cells were evaluated. First, randomly selected GFP-positive or control cells were manually encircled with the Freehand selection tool, and then the selected area was analyzed in the channel of interest (Analyze Particles, Show, Masks), and the count and average size (in pixel^2^) were noted from the summary of masked images. At least 10 images from 4–6 animals were evaluated for each genotype. These number/size data were processed as in the case of mutants, except that GFP-positive cells were compared to controls cells in the same image, and data was also normalized to cell size. Two-tailed two-sample unequal Student’s t test was used to estimate p values in all cases.

### Transmission Electron Microscopy

Larvae of the genotypes hs-Flp; UAS-p62-GFP/+; Act>CD2>Gal4, UAS-Dcr2/+ or hs-Flp; UAS-Dcr2/+; Act>CD2>Gal4, UAS-GFPnls/UAS-Atg4[C98A] or hs-Flp; UAS-Dcr2/+; Act>CD2>Gal4, UAS-GFPnls, r4-mCherry-Atg8a/UAS-Atg4[C98A] were heat shocked for 1 hour at 37°C to activate Act>CD2>Gal4 expression by hs-Flp in all fat body cells. Larvae were processed for electron microscopy 24 hours later as described previously. [16] Ultrathin sections were prepared from two animals per genotype and images were captured using a JEOL JEM-1011 transmission electron microscope with an Olympus Morada 11 megapixel camera and iTEM software (Olympus).

## Supporting Information

Figure S1 **Endogenous p62 dots in Atg mutants.** Loss of Atg2 (A), Atg7 (B), Atg8a (C), Atg13 (D), Atg18a (E) and Vps34 (F) all increase p62 aggregation. Scalebar in panel A equals 30 µm for all images. *Genotypes are: (A) Atg2[EP3697]/Df(3L)BSC119]; (B) Atg7[d77]/Atg7[d14]; (C) Atg8a[d4]; (D) Atg13[*Δ*81]; (E) Atg18a[KG03090]/Df(3L)Exel6112]; (F) Vps34[*Δ*m22].*
(TIF)

Figure S2 **The effect of additional RNAi and overexpression lines on p62 aggregation.** The effect of overexpressed dominant-negative Atg4 (B), dominant-negative Vps34 (G), wild-type Rheb (H), and knockdown of Atg2 (A), Atg7 (C), Atg8a (D), Atg9 (E), Atg14 (F), Atg16 (I, J), Atg18a (K, L), Pten (M, N) and Atms (O) on p62 aggregation. Scalebar in panel A equals 40 µm for all images. *Genotypes are: (A) hsFlp; UAS-Dcr2/+; Act>CD2>Gal4, UAS-GFPnls/Atg2[JF02786]; (B) hsFlp; UAS-Dcr2/+; Act>CD2>Gal4, UAS-GFPnls/UAS-Atg4[C98A]; (C) hsFlp; UAS-Dcr2/+; Act>CD2>Gal4, UAS-GFPnls/Atg7[JF02787]; (D) hsFlp; UAS-Dcr2/Atg8a[KK109654]; Act>CD2>Gal4, UAS-GFPnls/+; (E) hsFlp; UAS-Dcr2/+; Act>CD2>Gal4, UAS-GFPnls/Atg9[JF02891]; (F) hsFlp; UAS-Dcr2/Atg14[KK108559]; Act>CD2>Gal4, UAS-GFPnls/+; (G) hsFlp; UAS-Dcr2/UAS-Vps34[KD]; Act>CD2>Gal4, UAS-GFPnls/+; (H) hsFlp; UAS-Dcr2/+; Act>CD2>Gal4, UAS-GFPnls/UAS-Rheb[EP50.084cre(w-)]; (I) hsFlp; UAS-Dcr2/Atg16[KK105993]; Act>CD2>Gal4, UAS-GFPnls/+; (J) hsFlp; UAS-Dcr2/+; Act>CD2>Gal4, UAS-GFPnls/Atg16[HMS01347]; (K) hsFlp; UAS-Dcr2/Atg18a[KK105366]; Act>CD2>Gal4, UAS-GFPnls/+; (L) hsFlp; UAS-Dcr2/+; Act>CD2>Gal4, UAS-GFPnls/Atg18a[JF02898]; (M) hsFlp; UAS-Dcr2/Pten[KK101475]; Act>CD2>Gal4, UAS-GFPnls/+; (N) hsFlp; UAS-Dcr2/+; Act>CD2>Gal4, UAS-GFPnls/Pten[JF01987]; (O) hsFlp; UAS-Dcr2/Atms[GD20876]; Act>CD2>Gal4, UAS-GFPnls/+.*
(TIF)

Figure S3 **Knockdown of RpS8 or Atms inhibits starvation-induced mCherry-Atg8a and Lysotracker puncta formation.** Silencing of RpS8 or Atms strongly interferes with starvation-induced mCherry-Atg8a dot formation (panels A and B, respectively) and blocks punctate Lysotracker staining (panels C and D, respectively). Scalebar in panel A equals 30 µm for all images. *Genotypes are: (A) hs-Flp; UAS-Dcr2/RpS8[KK106835]; Act>CD2>Gal4, UAS-GFPnls, r4-mCherry-Atg8a/+; (B) hs-Flp; UAS-Dcr2/Atms[GD20876]; Act>CD2>Gal4, UAS-GFPnls, r4-mCherry-Atg8a/+; (C) hs-Flp; UAS-LampGFP/RpS8[KK106835]; Act>CD2>Gal4, UAS-Dcr2; (D) hs-Flp; UAS-LampGFP/Atms[GD20876]; Act>CD2>Gal4, UAS-Dcr2.*
(TIF)

Figure S4 **p62-GFP aggregation in additional RNAi and overexpression lines.** Knockdown of Atg7 (A), Atg8a (B), Atg9 (C), Atg14 (D), Atg18a (E), Pten (G), Tsc2 (H), RpS8(I), Atms (J) and expression of dominant-negative Vps34 (F) in p62-GFP expressing cell clones. Exposure times are indicated in the top right corner for each image. Scalebar in panel A equals 30 µm for all images. *Genotypes are: (A) hsFlp; UAS-p62-GFP/+; Act>CD2>Gal4, UAS-Dcr2/Atg7[JF02787]; (B) hsFlp; UAS-p62-GFP/Atg8a[KK109654]; Act>CD2>Gal4, UAS-Dcr2/+; (C) hsFlp; UAS-p62-GFP/+; Act>CD2>Gal4, UAS-Dcr2/Atg9[JF02891]; (D) hsFlp; UAS-p62-GFP/Atg14[KK108559]; Act>CD2>Gal4, UAS-Dcr2/+; (E) hsFlp; UAS-p62-GFP/Atg18a[KK105366]; Act>CD2>Gal4, UAS-Dcr2/+; (F) hsFlp; UAS-p62-GFP/UAS-Vps34[KD]; Act>CD2>Gal4, UAS-Dcr2/+; (G) hsFlp; UAS-p62-GFP/+; Act>CD2>Gal4, UAS-Dcr2/Pten[JF01987]; (H) hsFlp; UAS-p62-GFP/TSC2[KK103417]; Act>CD2>Gal4, UAS-Dcr2/+; (I) hsFlp; UAS-p62-GFP/RpS8[KK106835]; Act>CD2>Gal4, UAS-Dcr2/+; (J) hsFlp; UAS-p62-GFP/Atms[GD20876]; Act>CD2>Gal4, UAS-Dcr2/+.*
(TIF)

Figure S5 **The effect of Atg16 RNAi lines on starvation-induced punctate Lysotracker staining in L3 and L2 larval stages.** Both RNAi lines for Atg16 show a similar block of Lysotracker puncta formation in L3 (panels A and C), while the size of these dots is only reduced significantly by Atg16^HMS^ in L2 (compare D to B; see also panel E for statistics). * indicates a significant difference (p<0.05), ** indicates a very significant difference (p<0.01), based on two-tailed two-sample unequal Student’s t tests. Scalebar in panel A equals 30 µm for panels A, C, and scalebar in panel B equals 30 µm for panels B, D. *Genotypes are: (A,B) hs-Flp; UAS-LampGFP/Atg16[KK105993]; Act>CD2>Gal4, UAS-Dcr2; (C,D) hs-Flp; UAS-LampGFP/+; Act>CD2>Gal4, UAS-Dcr3/Atg16[HMS01347].*
(TIF)

Figure S6 **Overexpression of mCherry-Atg8a rescues the effect of Atg8a mutation or expression of dominant-negative Atg4 on p62 accumulation.** Expression of mCherry-Atg8a reduces the size and number of p62 aggregates in Atg8a null mutants (A; see panel B for statistics). No accumulation of p62 dots is observed in fat body cells coexpressing mCherry-Atg8a and dominant-negative Atg4 relative to control cells (C; see panel D for statistics – no significant difference is seen). Scalebar in panel A equals 30 µm. *Genotypes are: (A)Atg8a[d4]; +/+; r4-mCherry-Atg8a/+; (B) hs-Flp; UAS-Dcr2/+; Act>CD2>Gal4, UAS-GFPnls, r4-mCherry-Atg8a/UAS-Atg4[C98A].*
(TIF)
