# Supplementary figures and images for: Advantages and Limitations of Different p62-Based Assays for Estimating Autophagic Activity in Drosophila
Source: PLoS One. 2012 Aug 31;7(8):e44214. doi: 10.1371/journal.pone.0044214 (PMC3432079; doi:10.1371/journal.pone.0044214)

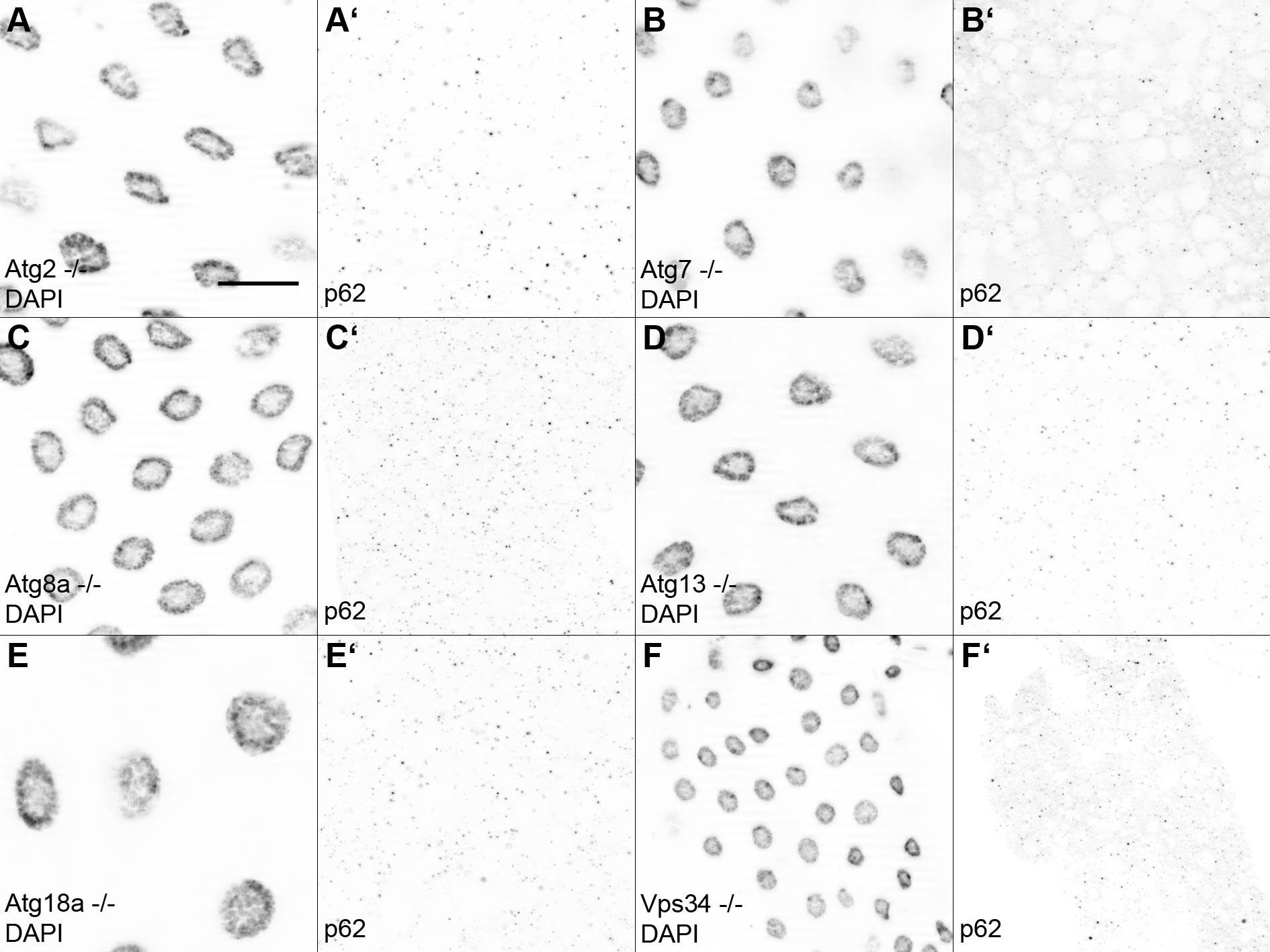

Supplement: Figure S1 — Endogenous p62 dots in Atg mutants. Loss of Atg2 (A), Atg7 (B), Atg8a (C), Atg13 (D), Atg18a (E) and Vps34 (F) all increase p62 aggregation. Scalebar in panel A equals 30 µm for all images. Genotypes are: (A) Atg2[EP3697]/Df(3L)BSC119]; (B) Atg7[d77]/Atg7[d14]; (C) Atg8a[d4]; (D) Atg13[Δ81]; (E) Atg18a[KG03090]/Df(3L)Exel6112]; (F) Vps34[Δm22]. (TIF) [file pone.0044214.s001.tif]

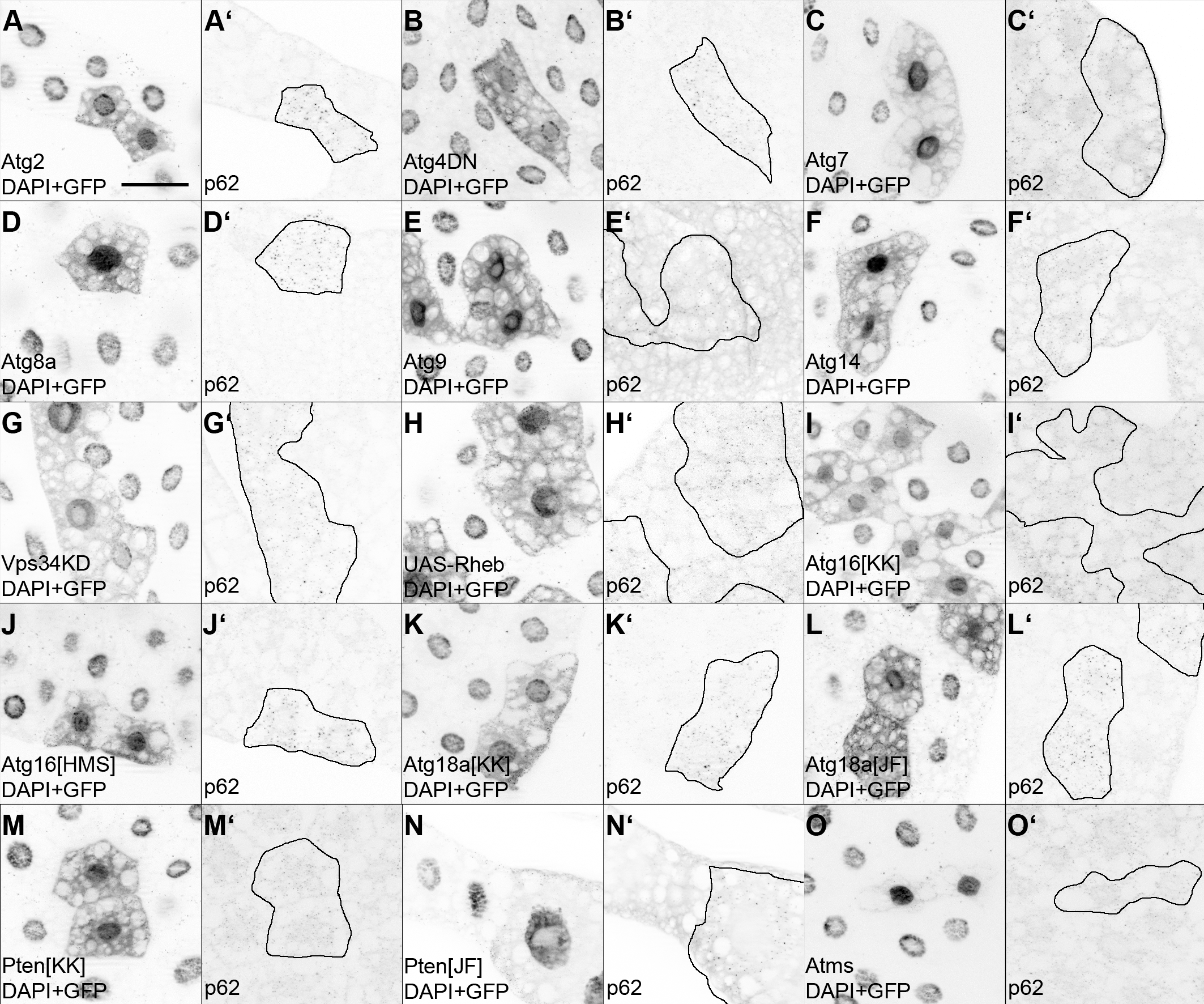

Supplement: Figure S2 — The effect of additional RNAi and overexpression lines on p62 aggregation. The effect of overexpressed dominant-negative Atg4 (B), dominant-negative Vps34 (G), wild-type Rheb (H), and knockdown of Atg2 (A), Atg7 (C), Atg8a (D), Atg9 (E), Atg14 (F), Atg16 (I, J), Atg18a (K, L), Pten (M, N) and Atms (O) on p62 aggregation. Scalebar in panel A equals 40 µm for all images. Genotypes are: (A) hsFlp; UAS-Dcr2/+; Act>CD2>Gal4, UAS-GFPnls/Atg2[JF02786]; (B) hsFlp; UAS-Dcr2/+; Act>CD2>Gal4, UAS-GFPnls/UAS-Atg4[C98A]; (C) hsFlp; UAS-Dcr2/+; Act>CD2>Gal4, UAS-GFPnls/Atg7[JF02787]; (D) hsFlp; UAS-Dcr2/Atg8a[KK109654]; Act>CD2>Gal4, UAS-GFPnls/+; (E) hsFlp; UAS-Dcr2/+; Act>CD2>Gal4, UAS-GFPnls/Atg9[JF02891]; (F) hsFlp; UAS-Dcr2/Atg14[KK108559]; Act>CD2>Gal4, UAS-GFPnls/+; (G) hsFlp; UAS-Dcr2/UAS-Vps34[KD]; Act>CD2>Gal4, UAS-GFPnls/+; (H) hsFlp; UAS-Dcr2/+; Act>CD2>Gal4, UAS-GFPnls/UAS-Rheb[EP50.084cre(w-)]; (I) hsFlp; UAS-Dcr2/Atg16[KK105993]; Act>CD2>Gal4, UAS-GFPnls/+; (J) hsFlp; UAS-Dcr2/+; Act>CD2>Gal4, UAS-GFPnls/Atg16[HMS01347]; (K) hsFlp; UAS-Dcr2/Atg18a[KK105366]; Act>CD2>Gal4, UAS-GFPnls/+; (L) hsFlp; UAS-Dcr2/+; Act>CD2>Gal4, UAS-GFPnls/Atg18a[JF02898]; (M) hsFlp; UAS-Dcr2/Pten[KK101475]; Act>CD2>Gal4, UAS-GFPnls/+; (N) hsFlp; UAS-Dcr2/+; Act>CD2>Gal4, UAS-GFPnls/Pten[JF01987]; (O) hsFlp; UAS-Dcr2/Atms[GD20876]; Act>CD2>Gal4, UAS-GFPnls/+. (TIF) [file pone.0044214.s002.tif]

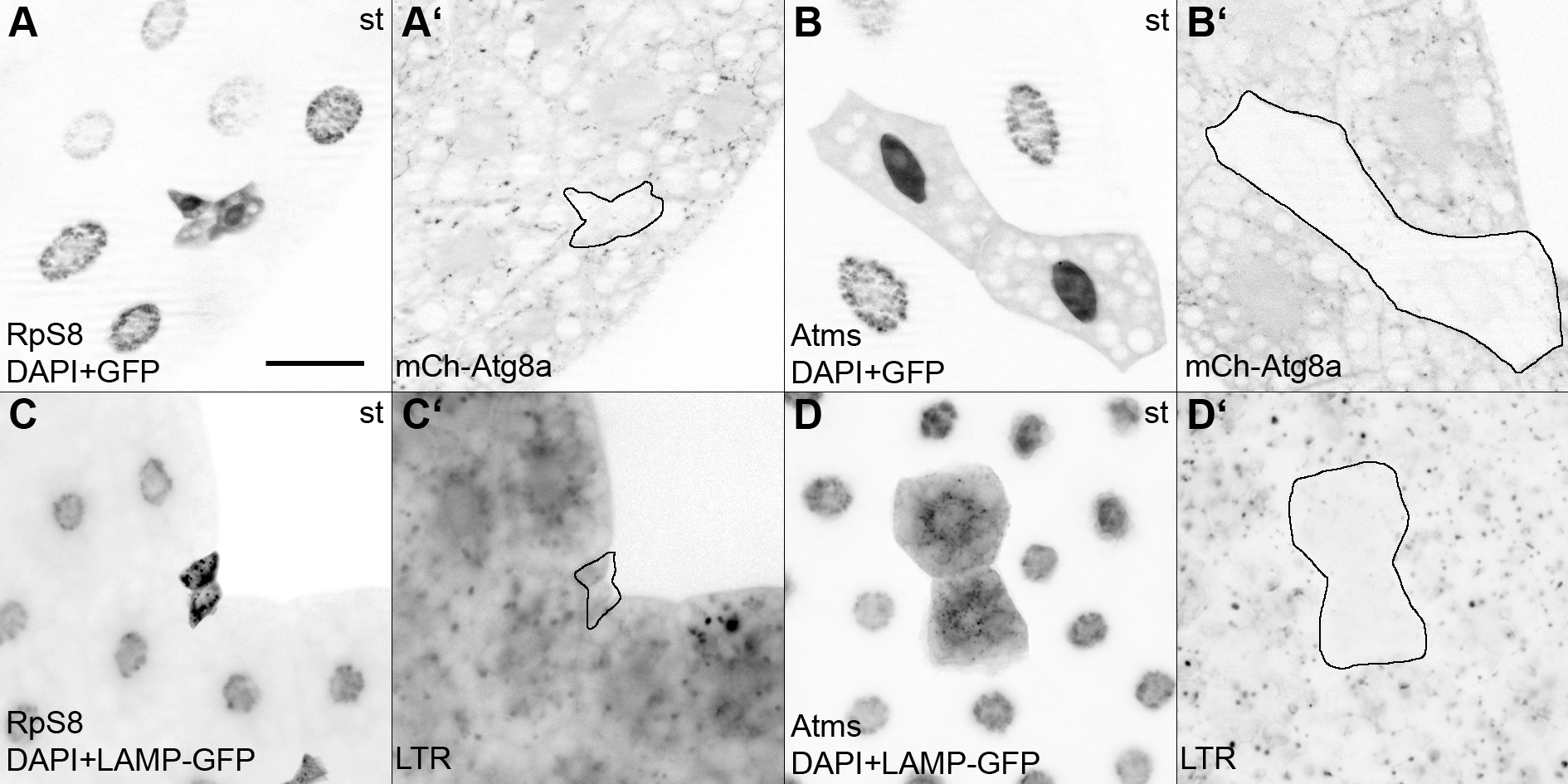

Supplement: Figure S3 — Knockdown of RpS8 or Atms inhibits starvation-induced mCherry-Atg8a and Lysotracker puncta formation. Silencing of RpS8 or Atms strongly interferes with starvation-induced mCherry-Atg8a dot formation (panels A and B, respectively) and blocks punctate Lysotracker staining (panels C and D, respectively). Scalebar in panel A equals 30 µm for all images. Genotypes are: (A) hs-Flp; UAS-Dcr2/RpS8[KK106835]; Act>CD2>Gal4, UAS-GFPnls, r4-mCherry-Atg8a/+; (B) hs-Flp; UAS-Dcr2/Atms[GD20876]; Act>CD2>Gal4, UAS-GFPnls, r4-mCherry-Atg8a/+; (C) hs-Flp; UAS-LampGFP/RpS8[KK106835]; Act>CD2>Gal4, UAS-Dcr2; (D) hs-Flp; UAS-LampGFP/Atms[GD20876]; Act>CD2>Gal4, UAS-Dcr2. (TIF) [file pone.0044214.s003.tif]

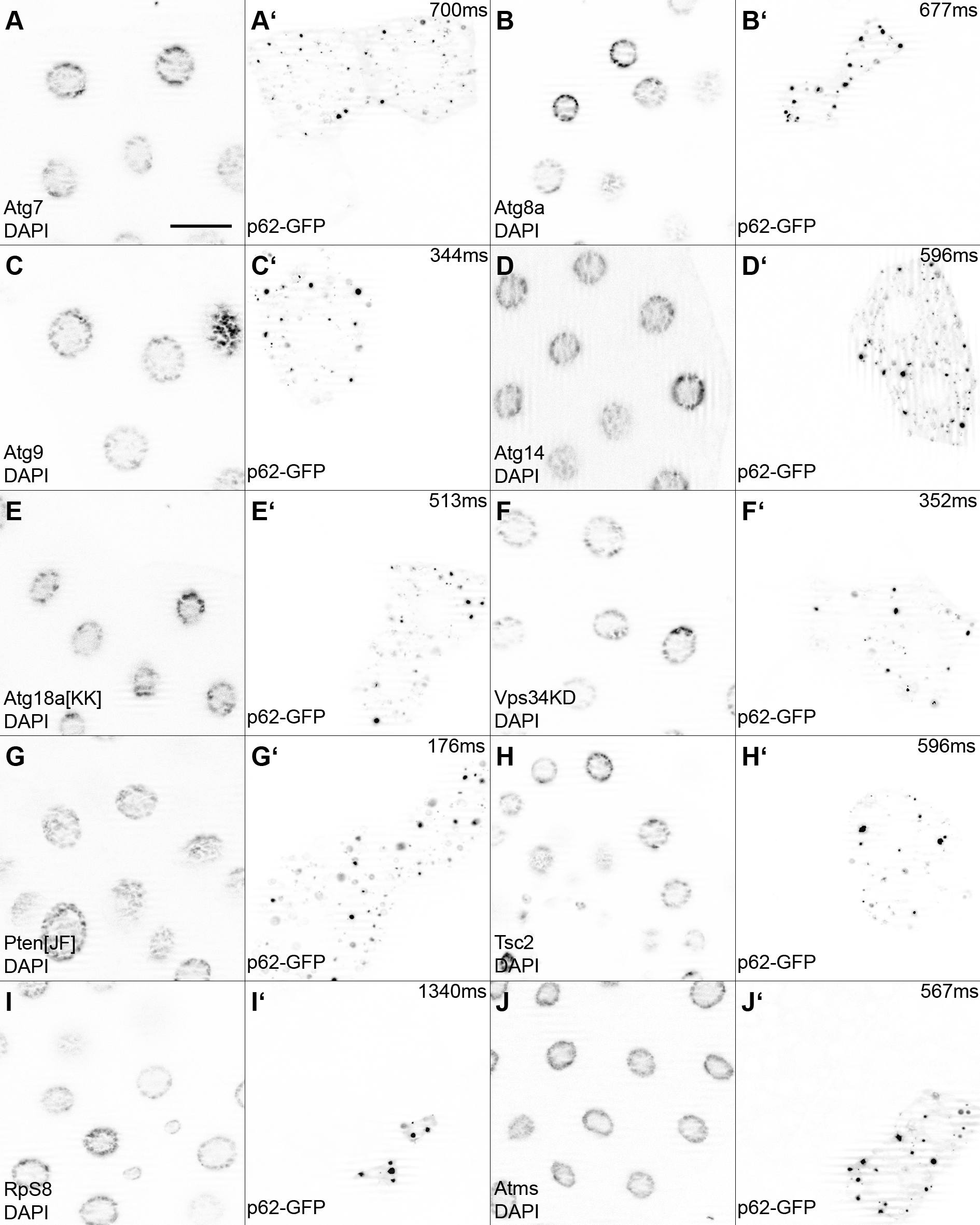

Supplement: Figure S4 — p62-GFP aggregation in additional RNAi and overexpression lines. Knockdown of Atg7 (A), Atg8a (B), Atg9 (C), Atg14 (D), Atg18a (E), Pten (G), Tsc2 (H), RpS8(I), Atms (J) and expression of dominant-negative Vps34 (F) in p62-GFP expressing cell clones. Exposure times are indicated in the top right corner for each image. Scalebar in panel A equals 30 µm for all images. Genotypes are: (A) hsFlp; UAS-p62-GFP/+; Act>CD2>Gal4, UAS-Dcr2/Atg7[JF02787]; (B) hsFlp; UAS-p62-GFP/Atg8a[KK109654]; Act>CD2>Gal4, UAS-Dcr2/+; (C) hsFlp; UAS-p62-GFP/+; Act>CD2>Gal4, UAS-Dcr2/Atg9[JF02891]; (D) hsFlp; UAS-p62-GFP/Atg14[KK108559]; Act>CD2>Gal4, UAS-Dcr2/+; (E) hsFlp; UAS-p62-GFP/Atg18a[KK105366]; Act>CD2>Gal4, UAS-Dcr2/+; (F) hsFlp; UAS-p62-GFP/UAS-Vps34[KD]; Act>CD2>Gal4, UAS-Dcr2/+; (G) hsFlp; UAS-p62-GFP/+; Act>CD2>Gal4, UAS-Dcr2/Pten[JF01987]; (H) hsFlp; UAS-p62-GFP/TSC2[KK103417]; Act>CD2>Gal4, UAS-Dcr2/+; (I) hsFlp; UAS-p62-GFP/RpS8[KK106835]; Act>CD2>Gal4, UAS-Dcr2/+; (J) hsFlp; UAS-p62-GFP/Atms[GD20876]; Act>CD2>Gal4, UAS-Dcr2/+. (TIF) [file pone.0044214.s004.tif]

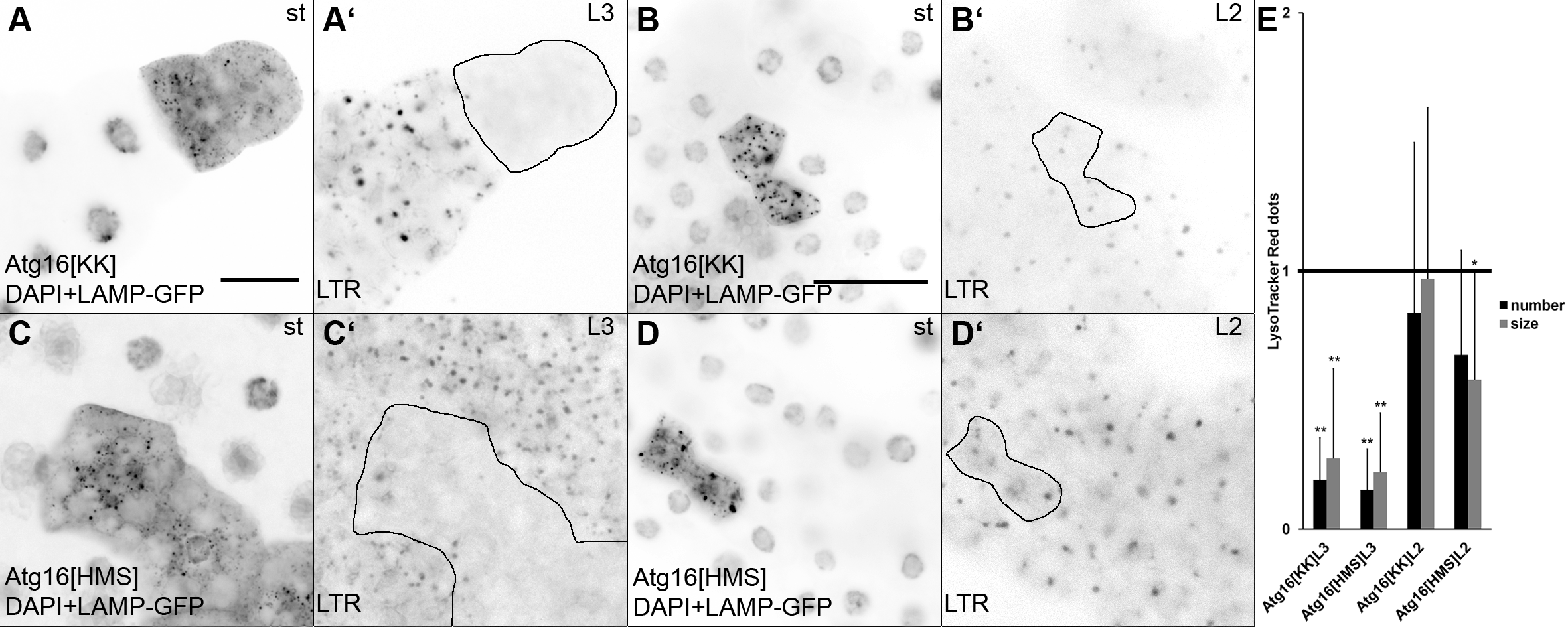

Supplement: Figure S5 — The effect of Atg16 RNAi lines on starvation-induced punctate Lysotracker staining in L3 and L2 larval stages. Both RNAi lines for Atg16 show a similar block of Lysotracker puncta formation in L3 (panels A and C), while the size of these dots is only reduced significantly by Atg16HMS in L2 (compare D to B; see also panel E for statistics). * indicates a significant difference (p<0.05), ** indicates a very significant difference (p<0.01), based on two-tailed two-sample unequal Student’s t tests. Scalebar in panel A equals 30 µm for panels A, C, and scalebar in panel B equals 30 µm for panels B, D. Genotypes are: (A,B) hs-Flp; UAS-LampGFP/Atg16[KK105993]; Act>CD2>Gal4, UAS-Dcr2; (C,D) hs-Flp; UAS-LampGFP/+; Act>CD2>Gal4, UAS-Dcr3/Atg16[HMS01347]. (TIF) [file pone.0044214.s005.tif]

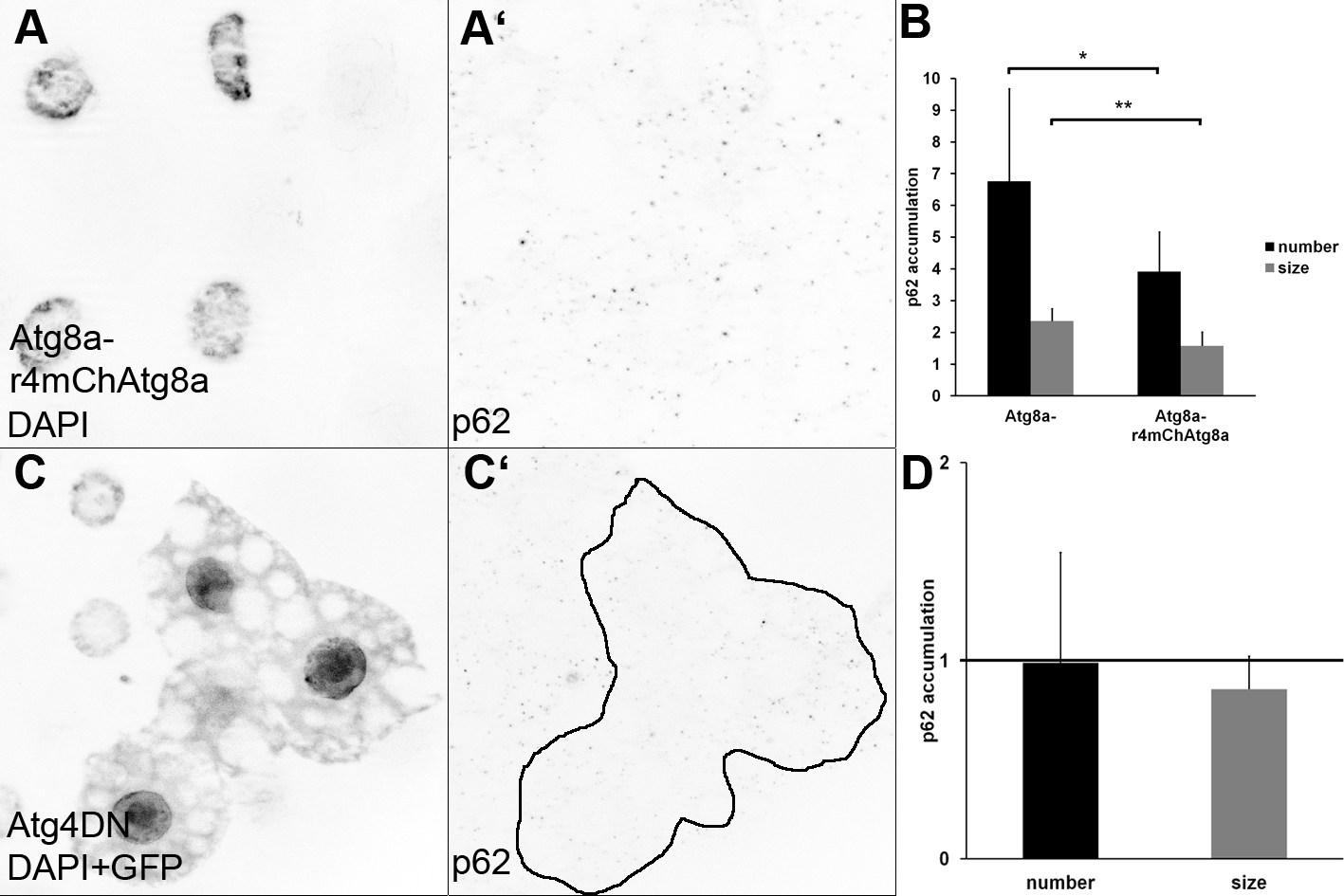

Supplement: Figure S6 — Overexpression of mCherry-Atg8a rescues the effect of Atg8a mutation or expression of dominant-negative Atg4 on p62 accumulation. Expression of mCherry-Atg8a reduces the size and number of p62 aggregates in Atg8a null mutants (A; see panel B for statistics). No accumulation of p62 dots is observed in fat body cells coexpressing mCherry-Atg8a and dominant-negative Atg4 relative to control cells (C; see panel D for statistics – no significant difference is seen). Scalebar in panel A equals 30 µm. Genotypes are: (A)Atg8a[d4]; +/+; r4-mCherry-Atg8a/+; (B) hs-Flp; UAS-Dcr2/+; Act>CD2>Gal4, UAS-GFPnls, r4-mCherry-Atg8a/UAS-Atg4[C98A]. (TIF) [file pone.0044214.s006.tif]
